# Feeling for the Other With Ease: Prospective Actors Show High Levels of Emotion Recognition and Report Above Average Empathic Concern, but Do Not Experience Strong Distress

**DOI:** 10.3389/fpsyg.2021.543846

**Published:** 2021-06-01

**Authors:** Isabell Schmidt, Tuomas Rutanen, Roberto S. Luciani, Corinne Jola

**Affiliations:** Division of Psychology and Forensic Sciences, School of Applied Sciences, Abertay University, Dundee, United Kingdom

**Keywords:** performing arts, emotional resilience, cognitive empathy, dance, theatre, psychology, social interaction, perspective taking

## Abstract

Differences in empathic abilities between acting, dance, and psychology students were explored, in addition to the appropriateness of existing empathy measures in the context of these cohorts. Students (*N* = 176) across Higher Education Institutions in the United Kingdom and Europe were included in the online survey analysis, consisting of the Reading the Mind in the Eyes (RME) test, the Interpersonal Reactivity Index (IRI), the Empathy Quotient (EQ), and the E-drawing test (EDT), each measuring particular facets of empathy. Based on existing evidence and our understanding of the discipline practices, we predicted that acting students would perform the best at identifying people’s emotional expressions but might lack other cognitive or affective empathy skills, particularly those related to emotional reactions. This cohort thus provides an opportunity to evaluate different empathy measures. While actors showed significantly higher RME scores than dancers, the difference between actors and psychologists was marginal. Moreover, actors’ scores did not differ significantly on other empathy measures, such as their concern for others’ emotional wellbeing or fantasy, both measured by IRI subscales. Psychology students scored highest in the IRI perspective taking subscale and the data supported anecdotal evidence that psychologists were more concerned for others’ emotional wellbeing than dancers or actors. Dancers seemed the least concerned with others’ perspectives and emotional states, which we explained through a somatosensory ‘inward’ focus required by their art form. Nevertheless, compared to the general population, our groups reported higher empathic abilities on all IRI subscales except for personal distress. Altogether, our study shows that the RME, the IRI, and the EDT vary in their susceptibility to different facets of empathic abilities in acting, dance, and psychology students whereas the EQ does not. Emotions can be expressed and perceived through language, facial expressions, or behavior. As many empathy tests focus on one type of signal they might miss other strategies. Where empathy tests are applied to individuals that have a predominance to read or respond to others in a particular way, as we showed through these three disciplines, they might not capture these empathic strategies. We thus propose that empathy tests must evolve by means of integrating varied forms of communication.

## Introduction

Empathy is understood to describe the ability to recognize the emotional and mental states of others and can involve the sharing of their feelings or be limited to a cognitive understanding (e.g., Decety and Meyer, 2008). Empathy is crucial for social interaction. Indeed, when empathic abilities are impaired, such as in severe forms of autism spectrum disorders, social interactions are difficult (DSM-IV-TR, American Psychiatric Association, 2000). Whilst inter-individual differences in some forms of empathy may be stable (Leiberg and Anders, 2006), there is strong evidence for possible improvements (e.g., Lam et al., 2011). Empathic abilities of individuals on the spectrum, for example, have been targeted successfully through specific training from theater and physical practices, such as participatory play and theater interventions (Corbett et al., 2016; Beadle-Brown et al., 2018) or dance and yoga (Koehne et al., 2016a; Litchke et al., 2018, respectively). Moreover, theoretical- and knowledge-based empathy training forms were also found to be effective for this cohort (Beaumont and Sofronoff, 2008; Holopainen et al., 2019). These intervention choices are not by coincidence. Among various artistic and academic disciplines, three stand out as especially focused on empathic processes: acting, dance, and psychology. In each practice, empathy plays a pivotal and distinct role; achieved through the use of language, the body and the mind.

Theater is particularly involved with the complexities of human emotions and reasoning (Noice and Noice, 2013; Gentzler et al., 2020), which are the cornerstones of empathy (Decety and Meyer, 2008). Moreover, feeling with or for actors is recognized as a part of theater audiences’ engagement (McConachie, 2008). In dance, audiences report experiencing empathy as well as cognitive reasoning, varying not only in form but also in intensity, based on their personal background (Reason and Reynolds, 2010). This is in line with the observation that the level of sensorimotor engagement of visually experienced dance spectators watching their preferred style of dance correlates with their ability to fantasize, a specific facet of empathy (Jola et al., 2012). Finally, psychologists, both practice-based and research-based, must understand how other people feel in order to draw valid conclusions (Elliott et al., 2018). In the human service and social science disciplines, didactic empathy training is most popular and highly effective (Lam et al., 2011). It is therefore reasonable to suggest that individuals with different practical and cultural experiences show distinctive empathic patterns. Yet, if all three disciplines employ empathy in various forms, its definition becomes unclear. Indeed, despite ongoing extensive research into empathy, there is a lack of agreement on its theoretical conceptualization.

Whether basic empathic abilities are inborn or not, whether empathy is one concept that entails several dimensions, or whether empathy solely compromises affective components, is still debated (Heyes, 2018). The review of Cuff et al. (2016) concluded that there is increasing evidence to suggest that affective and cognitive empathy are two separate although complementary constructs. Accordingly, affective empathy refers to the person’s experience of emotion as evoked by an external emotional stimulus and cognitive empathy refers to the ability to understand the feelings of another person. Thus forth, several authors situate this latter ability within the concept of theory of mind, whereby one mentalises about others’ states, thoughts, and feelings without being emotionally affected by them (e.g., Blair, 2005; Goldstein and Winner, 2012). Nevertheless, theory of mind and empathy rely on overlapping neuronal processes (Völlm et al., 2006). Moreover, whilst some authors propose that motor simulation is required for empathy in order to experience someone else’s emotions (Thioux and Keysers, 2010), others suggest that empathy is predominantly an affective experience, located in the insula (Bernhardt and Singer, 2012), or can in fact rely on cognitive understanding alone (Hodges and Myers, 2007). In addition, authors who discuss embodied accounts of empathy propose that empathy has a proprioceptive dimension, whereby kinaesthetic empathy describes the ability to experience empathy in response to merely observing movement, such as when watching a dance performance (Reason and Reynolds, 2010). Moreover, at times, even the form of measurement itself is used to define the type of empathy, as in behavioral empathy (Cuff et al., 2016; Teding van Berkhout and Malouff, 2016). Consequently, depending on the tests used, different facets of empathy are assessed. Here, we consider empathy as a multidimensional construct and explore interindividual differences in acting, dance, and psychology students in their abilities across a multitude of facets assigned to empathy as assessed by a variety of tests. Notably, to this day, the question has remained open as to which measurement reflects empathic abilities most accurately while remaining easy and quick to administer.

The most widely used empathy tests are the Reading the Mind in the Eyes test (RME; Baron-Cohen et al., 2001) measuring primarily cognitive empathic ability and the Interpersonal Reactivity Index (IRI; Davis, 1983) measuring also other empathy facets. Another commonly used measure is the self-reported Empathy Quotient (EQ; Baron-Cohen and Wheelwright, 2004), which provides an overall score of empathy without dividing between affective and cognitive empathy. There are many more empathy measures in use, such as the Questionnaire Measure of Emotional Empathy (Mehrabian and Epstein, 1972) or the Toronto Empathy Questionnaire (Spreng et al., 2009). Notably, Yu and Kirk (2009) identified 20 different empathy measurement tools in the nursing literature over two decades alone. Here, we focus on the RME, IRI, and the EQ plus the less commonly used E-drawing task (EDT) because over the years, they have been repeatedly used in acting, dance, and psychology studies with each measure arguably addressing specific aspects prevalent in these three disciplines.

The RME consists of a set of pictures of the eye regions, taken from Hollywood films, and participants are asked to identify from a selection of four emotions, which one is expressed in the still. The revised version of the RME is considered a valid measurement focusing on social intelligence and social sensitivity (Baron-Cohen et al., 2001). As the RME focuses on the recognition of emotional expressions in the eye region, it could be a useful test to differentiate individuals with a particular empathic faculty in recognizing facial expressions, such as actors, from individuals that excel in other forms of empathy, as for example measured by the IRI. The IRI examines empathy as a multidimensional construct and consists of four subscales, where empathic concern (IRI-EC) corresponds to the emotional or affective dimension of empathy or the feeling of warmth or compassion for others, while perspective taking (IRI-PT) and fantasy (IRI-FS) correspond to the cognitive dimension (Davis, 1983). Personal distress (IRI-PD) also corresponds to the emotional (or affective) dimension of empathy. In contrast to empathic concern, which refers to the feelings toward others, personal distress refers to a person’s own feelings of unease and anxiety in response to another’s negative experience (Davis, 1983). The IRI has been employed in all three disciplines most recently, not least because its broad approach to empathy (e.g., Poorman, 2009; Gujing et al., 2019; Panero and Winner, 2021). In fact, Baron-Cohen and Wheelwright (2004) developed the EQ in response to the criticism that the IRI may measure abilities broader than empathy, such as imagination and self-control. The final empathy measurement tool employed here, the EDT, is a measure of passive perspective taking, designed by Hass (1984). It is a very quick but less common measure in the realms of empathy. In the EDT, participants are asked to draw an E on their forehead, and the orientation they draw it in determines what perspective they were taking when performing the task. Drawing the E viewed from ‘within,’ renders the E mirrored for the outside viewer, and vice versa. Though perspective taking, as measured also by the IRI subscale, plays an important role in empathy as well as theory of mind, in the EDT, it is considered a form of empathy linked to active perspective taking and self-awareness (Hass, 1984). Active perspective taking was shown to encourage people to change deeply held beliefs, such as views on abortion (Tuller et al., 2015), and to stimulate short-term prosocial behavior (Adida et al., 2018). Whilst other perspective-taking measures have been criticized for requiring the experimenter to ask the participant directly to adopt other perspectives (Erle and Topolinski, 2017), this is not a problem the EDT does run into.

In general, objective measures (e.g., RME and EDT) are considered more reliable than self-reported measures (e.g., IRI and EQ). Whilst the RME is widely criticized for its gender bias, outdated images, and its use of static stimuli in a non-fixed world, it is together with the EDT considered as a naturalistic, intuitive measurement of empathic abilities. Therefore, the RME and EDT are of particular interest. The IRI, on the other hand, has been found to be useful as it allows to measure empathy by four subscales. Furthermore, earlier research has found it to be a reliable and sensitive measurement, which shows subtle differences in individual’s emphatic approaches (e.g., Jola et al., 2012). Thus, comparing the IRI with the RME, for example, one would expect a counselor to have excellent capacity to empathize without experiencing high levels of personal distress (i.e., IRI-PD). A similar prediction was confirmed for actors (Gentzler et al., 2020). In their study, acting majors were expected to show high abilities in recognizing the emotional expression of others as well as having high emotional regulatory skills. For the latter, only the ability to modulate emotions in intensity was significant, which may, however, be related to the experience of personal distress. This regulatory ability is important, considering that actors often perform contexts of conflict where they have to be able to dissociate the performed aggression from their own feelings (Berceanu et al., 2020). Lastly, evidence for outstanding empathic abilities in response to dance training are less consistent in regard to the IRI, with partial affirmation for enhanced scores in personal distress and perspective taking (Gujing et al., 2019). In order to assess whether these discipline specific observations are inconclusive based on the type of tests used, they can be complemented by other measures such as the EQ or EDT. Moreover, dance is infamous for its various styles and training techniques that pose very specific demands on the performer, which consequently affects empirical research (Christensen and Jola, 2015). To some degree, this is congruent in acting with its seemingly conflicting practices (Noice and Noice, 2013). In this context, it is important to note that when considering existing training regimes, the focus should not only be on whether empathy can be enhanced through specific interventions, but which facets of empathic abilities are responsive to which type of training.

To our knowledge, most empathic training interventions employ experiential training based on theater practices, such as role play (e.g., Brunero et al., 2010). This makes sense, based on the understanding that for theater audiences to have an emotional experience, actors presumably have to be experts in emotional expression and affective processing, both of which are elements of empathy. The assumption that actors have advanced empathic skills is partly based on actors’ applied acting training in techniques such as Method acting. This form of acting encourages actors to experience their characters’ emotions in real time and through actual memories (Goldstein and Bloom, 2011), thereby sensitizing and enhancing emotional experiences and responsiveness in practitioners. Another important aspect of actors’ training and performance is the ability to execute and retain movement. Importantly, movements in a play can be considered as a choreographical score, as they rarely match the content of the verbal material. Dance classes therefore constitute a part of actors’ training and many actors continue to engage in movement-related practices. Notably though, the focus of actors’ movement practice is generally not to achieve technical perfection of actions that can only be executed through highly skilled physical training (as in dance). The actors’ emphasis is on executing actions in an experiencing way that allows feelings to emerge spontaneously within the movements as well as to respond ‘naturally’ (see Noice and Noice, 2013). For this, actors engage in a multitude of acting methods and role-play exercises. Most of these focus on enhancing their awareness and control of their own emotional experiences and expressions, which allows actors to effectively inhabit the characters they are expected to perform and consequently enables them to affect the feelings of other actors and audiences (Lippi et al., 2016). Indeed, empathy training through role-play exercises found empathy improvements, such as in nursing students’ empathy skills (e.g., Bas-Sarmiento et al., 2017).

Empathy training through acting practices has been found to be effective across the development. Goldstein and Winner (2012), for example, found that in their first study, children’s participation in 1 h weekly improvisation games over 10 months enhanced their empathic abilities more compared to children who enrolled in visual arts classes, whereas the scores in theory of mind did not differ between the two groups. This finding corresponds to what would be expected by the two-system theory of empathy proposed by Heyes (2018), whereby empathy, comprising empathic understanding, involves controlled processing and thus develops later in life, whilst empathy often described as ‘emotional contagion’ operates automatically and develops early. Notably though, children in Goldstein and Winner’s (2012) were self-selecting the courses and the data was not distinguished between children who had previously engaged in acting classes from those who had signed up for the first time. It is thus possible that self-selection and previous experience contributed to their findings. Importantly, in their second study, the authors found an effect of theater training on theory of mind at later stages in life. Compared to the control cohort in the visual arts and music, High School acting students showed higher scores in theory of mind before the intervention and improved further after 10 months of intense acting training. One can thus conclude that not only is empathy malleable, but that engagement in acting training that often involves imitating others shows higher levels in theory of mind during adolescence and is therefore already present at the onset of training. In addition, these studies highlight that acting practice impacts facets of empathic abilities differently at different stages of their career. In considering the findings of Goldstein and Winner (2012), it is to be noted, however, that the authors employed the RME to measure theory of mind. Also, the authors did not acknowledge the different interrelated dimensions of empathy as suggested by recent research (e.g., Thompson et al., 2019; Decety, 2020) and herein, but were aligning the term theory of mind with cognitive empathy and considering the term empathy to affiliate closer to affective empathy. We thus predict that acting students may show a significant advantage in the RME, but not in other cognitive or affective empathy dimensions, such as measured by the IRI subscales.

Similar to actors, dancers need to be prolific in perceiving and expressing emotions. The difference is that dancers start their training at a young age in order to become experts in action execution and observation of body movements (Bläsing et al., 2012). It is unclear, however, whether their empathic abilities are related to their physical, observational, or communication abilities. Based on the genetic profile of dancers, Bachner-Melman et al. (2005) hypothesized that dancers’ phenotype is determined by social communication; over and above other characteristics related to sensorimotor execution. Others have suggested that dancers’ action observation and execution skills can be expected to be reflected in better recognition of mental, and especially emotional, states of others as well as better perspective taking skills (see Sevdalis and Keller, 2011). However, the argument exists that these arise primarily and preferably through somatic practice and enhanced embodied simulation (Batson, 2014). To add to the complexity, a study on Hip Hop dancers by Bonny et al. (2017) confirmed that dance experience was related to better recognition of facial emotion expressions as measured by the RME. Yet, Horwitz et al. (2015) found that whilst higher dance engagement is related with better communications of feelings with the environment, the identification of others’ emotions is not. Notably though, the authors employed a measurement out with the standard empathy measures, designed for assessing alexithymia, a construct that describes the difficulty in finding words for emotions and feelings.

Irrespective of the specific factors that may enhance empathic abilities through dancing, dancers’ skills in different facets of empathy are ambiguous, in particular their perspective taking skills. Dancers engage extensively in egocentric body transformation tasks during training. Egocentric body transformation is a cognitive process, whereby an individual mentally changes their own perspective without moving their physical position in space (Kessler and Thomson, 2010). This process takes place for example, when a dancer copies a movement from a teacher or dance partner. As empathic perspective taking is related to the tendency and ability to take on the position of another, it is plausible to assume and empirically supported by Erle and Topolinski (2015), that mechanisms involved in empathic perspective taking and egocentric body transformation are closely related. Hence, based on the frequent changes in perspective through embodiment, dancers are expected to outperform others on perspective taking tasks as was confirmed through a dance intervention in school children by Jansen et al. (2013). However, compared to non-athletes, dancers neither consistently show better perspective taking skills nor an enhanced object-based spatial manipulation performance (e.g., Jola and Mast, 2005; Voyer and Jansen, 2017). We thus predict that whilst dancers may perform better than actors on perspective taking skills through their extensive physical embodied practice, they are less skilled than psychologists, who have previously been found to excel in perspective taking tasks. Importantly, though, these latter studies employed cognitive perspective taking tests that do not directly relate to perspective taking as a facet of empathic ability. Moreover, the lack of dancers’ perspective taking skills might be related to the type of non-dynamic stimuli used. For instance, when non-professional dancers watch dance, depending on their frequency of dancing, their self-reported empathy scores are correlated with their accuracy in recognizing the emotion intensity expressed in dance movements in dynamic stimuli, but not in stills (Sevdalis and Keller, 2012).

It has also been suggested that the ability to mirror movements is linked to empathic behaviors (McGarry and Russo, 2011). Mirroring is indeed frequently practiced in dance training and dance movement therapy - a psychotherapeutic approach, yet statistical evidence of the effect of mirroring on empathy is mixed (Bekkali et al., 2020). Moreover, to our knowledge, there is no evidence of professional dancers themselves showing enhanced empathic abilities with the exception of one recent study by Gujing et al. (2019). The authors found higher self-reported empathic abilities in the form of perspective taking, personal distress, and empathic concern in the dance group compared to the control cohort, as measured by the IRI. The authors suggested that dance training enhanced neurofunctional connectivity, which facilitates the integration of intero-/exteroceptive information and results in better affective sensitivity. Yet, whilst this might underly individuals’ enhanced empathic abilities, the authors did not discuss that a higher PD score is not desirable. One could thus argue that dancers’ practice enhances their affective empathy indicated in empathic concern (IRI-EC) yet social interactions with distressed individuals affect their own emotional wellbeing (IRI-PD).

For psychologists, empathy is fundamental. Psychology students train to help others, regardless of the path they choose after graduating (Harton and Lyons, 2003). As a result, they are on average more empathic than students from other academic disciplines (Harton and Lyons, 2003). The importance of affective empathic abilities is particularly evident in psychologists, counselors, and across the healthcare sector, where a high level of empathic concern and perspective taking capacity is desired, while low levels of personal distress responses can be a means to manage one’s own mental health. As noted by Teding van Berkhout and Malouff (2016), studies show that higher empathic ability of health care professionals contributes to better outcome from the therapeutic interventions. Their meta-analysis confirmed that empathy training is particularly effective in health professionals and university students, whose training showed a larger effect size than that of children, teenagers, and other adults. Further studies suggest that even theoretical learning about the neurobiology of emotions can have a positive impact on patient-rated empathy ratings for physicians (Riess et al., 2012) and that empathy training enhances the professional-patient relationship and its ability to aid better diagnoses (Riess et al., 2012; Petrucci et al., 2016; Bas-Sarmiento et al., 2017). Considering that high empathic abilities are also related to better patient outcomes in physical and mental health (Gladstein and Feldstein, 1983; Hojat et al., 2011; Watson et al., 2014, respectively), the role of empathy training is crucial in medical as well as counseling professions.

Despite strong evidence for the effectiveness of empathy training through experiential practices (e.g., games or role-play), didactic-theoretical training, practical skills training, or a mix of these, it is evident from the above that the findings are mixed (e.g., Lam et al., 2011). Lam and co- authors emphasize in their review that whilst some success has been reported for increasing cognitive empathy and behavioral responses, the existing evidence is insufficient to conclude that the emotional elements of empathy were altered across different professions in the social science disciplines and human services. Here, we have three professional pathways, with a different emphasis on empathic skills and training forms: Theater (a mix of theoretical and practical skills training), dance (predominantly practical skills training), and psychology (in early years predominantly didactic skills training). Whilst existing literature has studied empathic abilities in each of these disciplines, to our knowledge no study has yet provided a comparison of a set of specific empathic abilities across these groups.

It is important to remember that firstly, as outlined above, the effectiveness of empathy training differs across different age groups with children benefitting more than adults do. Moreover, studies that tracked individuals’ progression in empathic abilities during their professional education found conflicting evidence (e.g., Andersen et al., 2020). Secondly, methods of professional training vary even within one profession. It is thus important to first understand whether aspects of empathic abilities differ before individuals intensively practice one particular method. Thirdly, as mentioned earlier, a particular challenge in comparing existing studies is that they all use different measures of empathic abilities and a more comprehensive test-battery is thus needed.

The present study therefore set out to investigate specific facets of empathic abilities in young adults that have engaged in some form of recreational empathy training during their adolescence, such as acting, dance, or psychology but that are at the starting point of their professional pathway. If indeed several types of empathy exist, as research suggest (e.g., Cuff et al., 2016), the RME, IRI, EQ, and EDT used in combination with each other, seem ideal to explore differences between groups. This is particularly pertinent for our prediction of the actors’ empathic abilities, which is that they differ for cognitive empathy and other facets of empathy. We thus recognized the need to compare individuals’ natural inclination for specific facets of empathy that might have attracted them to their field due to existing abilities. To our knowledge, no study has yet compared these differences in empathic abilities between actors, dancers, and psychologists at the start of their professional pathways.

Acting, dance, and psychology students are expected to differ between the types of empathic abilities that they bring to their course and therefore differ in their empathy test scores at the start of their training. Identifying empathic abilities between acting, dance, and psychology students will also highlight which empathy tests might be particularly sensitive to differences between these groups. For example, if the RME and the subcategories of the IRI are indeed measuring distinct empathic abilities as previously suggested, individuals that specialize in one of the three disciplines are expected to show unique patterns of empathy scores.

## Materials and Methods

### Participants

We targeted Higher Education Institutions (HEI) to participate in this study across the United Kingdom and abroad. Higher Education Institutions encompass Universities, Colleges, and profession-oriented applied institutions. They are governmentally accredited organizations that provide special education at higher, postsecondary, tertiary, or third-level education. Overall, we contacted over 40 individuals that were either personal contacts with links to HEI or administration personnel working at selected HEI (27 dance, 13 psychology, and 11 acting). Of those, HEI in the United Kingdom, France, Germany, Malta, Belgium, Switzerland, Netherlands, and Norway agreed to cooperate. Of those 17 responses, 6 HEI with the core discipline of dance, 4 acting and 2 psychology included our study into their curriculum, to ensure students’ full engagement with the questionnaires. Two HEI with dance and three with acting curricula sent the link out to students to complete in their own time which led to only a small number of participant responses (<10). All participants had the choice to complete the questionnaires in either English or French. This study was reviewed and approved by the School of Social Health and Sciences Research Ethics Committee, Abertay University and formed part of a larger survey. Participants could only take part if they provided informed consent to participate.

A total of 372 participants started the online survey. Of these, 215 competed at least one empathy measure (57.79%). We then removed participants who were not in their first year of a graduate degree course in acting, dance, or psychology, and only studied one of the three disciplines, leaving 208 participants. As all questionnaires were language based, we excluded 12 female and 6 male participants who identified as Dyslexic (2 acting students, 11 dance students, and 5 psychology students). A further 10 dancers’ special needs data points were missing, and we thus excluded these participants. Of all included participants, 38 identified as male and 138 as female. Four participants did choose not to identify as either male or female and were excluded for statistical reasons only; leaving 176 participants; i.e., 29 acting students (17 females; overall age 20.76 ± 2.70), 82 dance students (64 females, overall age = 20.39 ± 2.55) and 65 psychology students (57 females, age = 19.83 ± 3.53) in the dataset for the analyses. However, not all participants completed the full set of questionnaires.

All participants completed the RME with the exception of 28 dance students, for whom the test failed due to an institutional firewall protection. After criteria-based exclusion, the EDT was completed by 165 participants (29 acting, 73 dance, and 63 psychology), the IRI by 138 (20 acting, 60 dance, and 58 psychology) and the EQ by 104 (12 acting, 44 dance, and 48 psychology). We then removed outliers for all measures within each discipline according to the interquartile range procedure (1.5 × IQR) with a maximum of three iterations, which identified five cases in RME (2 acting ≤ 20, and 3 psychology ≤ 19). For EQ, we identified one psychology case (≤ 24). See Table 1 for the final set of scores and participant numbers across the measures, discipline and gender (see Table 1).

**TABLE 1:** Average scores ± Standard Deviation for each test after outlier removals.

	**Acting**	**Dance**	**Psychology**	**Overall**
**E-drawing “E”**				
Males	50.00% (12)	47.06% (17)	0.00% (7)	38.89% (36)
Females	23.53% (17)	50.00% (56)	33.93% (56)	30.91% (129)
Total	34.48% (29)	49.32% (73)	30.16% (63)	39.39% (165)
**RME**				
Males	27.68 ± 2.33 (11)	22.90 ± 3.41 (10)	27.29 ± 3.25 (7)	25.50 ± 3.24 (28)
Females	26.73 ± 2.05 (16)	26.50 ± 3.20 (44)	27.36 ± 2.93 (55)	27.08 ± 2.98 (115)
Total	27.30 ± 2.23 (27)	25.83 ± 3.41 (54)	27.35 ± 2.94 (62)	26.77 ± 3.08 (143)
**IRI**				
Males	65.50 ± 12.47 (8)	60.60 ± 8.73 (10)	62.63 ± 8.80 (8)	62.73 ± 9.85 (26)
Females	72.50 ± 13.11 (12)	71.12 ± 11.00 (50)	77.02 ± 11.37 (50)	73.90 ± 11.64 (112)
Total	69.70 ± 13.00 (20)	69.37 ± 11.30 (60)	75.03 ± 12.07 (58)	71.80 ± 12.11 (138)
**EQ**				
Males	44.75 ± 17.54 (4)	40.89 ± 8.43 (9)	43.50 ± 6.98 (6)	42.52 ± 9.96 (19)
Females	39.88 ± 14.07 (8)	45.31 ± 11.43 (35)	47.73 ± 7.42 (41)	45.98 ± 10.10 (84)
Total	41.50 ± 14.68 (12)	44.41 ± 10.95 (44)	47.19 ± 7.43 (47)	45.34 ± 10.12 (103)

*Participant numbers are provided in brackets. Drawing E are the percentage and counts as in drawing the E in form of ‘E,’ so that the signer can read it.*

### Questionnaires and Procedure

Demographic information was collected first (i.e., information on participants’ education, age, gender, and training). Thereafter, we asked participants to respond to a context-based questionnaire (not included here) before completing four empathy measurements, namely, the E-drawing test (EDT), the Reading the Mind in the Eyes test (RME), the Interpersonal Reactivity Index (IRI), and finally the Empathy Quotient (EQ).

In the EDT, participants were asked to draw an E on their forehead (with their index finger or a pen which was randomly assigned), and report in which way it had been drawn (readable for themselves or for another person facing them). Since the EDT is an unusual test and there is a tendency to not do it but instead click to the next slide. Before being asked to assess the direction of their E with the help of two example images, we thus had in in-between section were we asked participants for their compliance and only continue to the next page if they have done so.

In the 36-item revised RME test (Baron-Cohen et al., 2001) participants were asked to look at pictures of eyes from film excerpts and identify which emotional expression is shown by selecting one out of four emotion descriptions. Participants were provided with two exemplars before starting the RME proper. In addition, participants had the option to check the meaning of the words on a separate glossary of all mental state terms, available via a web-link.

The next questionnaire was the 28-item Interpersonal reactivity index (IRI, Davis, 1980) which measured empathy on four subscales of perspective taking (IRI-PT), empathic concern (IRI-EC), personal distress (IRI-PD) and fantasy (IRI-FS) with seven questions for each subscale. IRI-PT was measured with questions such as “I believe that there are two sides to every question and try to look at them both” or in reversed forms such as “I sometimes find it difficult to see things from the “other guy’s” point of view.” Previously reported internal consistency for IRI-PT is α = 0.61 in males and α = 0.62 in females. The fantasy subscale entailed questions such as “After seeing a play or movie, I have felt as though I were one of the characters” or in reversed form “Becoming extremely involved in a good book or movie is somewhat rare for me” (Internal consistency was previously reported as α = 0.79 in males, and α = 0.81 in females). Personal distress was assessed through questions, such as “I tend to lose control during emergencies” or in a reversed form “When I see someone get hurt, I tend to remain calm” (Previously reported internal consistency in males was α = 0.68, and in females α = 0.76) and empathic concern with “I am often quite touched by things that I see happen” or in reversed form “Other people’s misfortunes do not usually disturb me a great deal” (Internal consistency reported previously was α = 0.72 in males and α = 0.70 in females).

The last questionnaire was the 40-item Empathy Quotient (EQ), which consisted of questions answered on a four-point Likert scale ranging from “strongly agree” to “strongly disagree” (Baron-Cohen and Wheelwright, 2004). The EQ was used to measure the state component of empathy with questions such as “I am quick to spot when someone in a group is feeling awkward or uncomfortable” and “I don’t tend to find social situations confusing” (Internal consistency was previously reported as α = 0.89) (Baron-Cohen and Wheelwright, 2004). According to the authors, the EQ is understood to measure the state of empathy well and is frequently employed because of its good validity. EQ was used to provide an overall score of empathy without diving between affective and cognitive empathy. The questionnaire was shortened to forty questions by removing the 20 control questions included in the original questionnaire.

### Analysis

Based on the number of dropouts across the survey, we calculated analyses for each empathy measure individually. Our main interest is on the effect of discipline. However, as it is well known that males and females differ in their empathic abilities, we first conducted univariate ANOVAs to confirm the validity of our dataset (i.e., that gender does show predicted effects). When there was no interaction between gender and discipline in the univariate ANOVA, we removed gender as a random effect from the model by running a mixed linear model based on Restricted Maximum Likelihood Estimate of Variance Component (REML) with the fixed effect discipline. For those analyses where discipline showed a strong trend for a significant effect, we conducted Bonferroni *post hoc t*-tests to identify differences between the three disciplines. A REML is preferable for unbalanced datasets like ours. Finally, as some of our participant numbers are low, we ran Bayesian ANOVA on JASP to verify the predictability strength of discipline. Finally, as we were interested in the relationships between the different tests, we ran correlation analyses across the empathy measures.

The IRI is regularly reported for the subscales. As these are of great value for our comparisons as discussed in the introduction, we conducted the analyses for each individual subscale. Lastly, the EDT test was analyzed using a chi-square, to evaluate whether the task completion varies for discipline and gender.

## Results

Descriptive data (Table 1) and inferential statistics for each empathy measure are reported in order of completion. The EDT was analyzed using Chi-Square which showed a very strong trend for a non-normal distribution for either drawing the E to be able to read by the signer (i.e., ‘mE’) or the mirror version of ‘E’ to be read by another across the disciplines, df(2), Pearson Chi-Square = 5.55, *p* = 0.062. In both, acting and psychology students, 2/3rd of participant draw the E so that the other can read it (65.5 and 69.8%, respectively) whereas only half of the dancers drew the E so that the other can read it (50.7%). The distribution across genders does not deviate from a normal distribution, Pearson Chi-Square = 0.005, *p* = 0.944.

Univariate ANOVA on RME scores with the between-subjects factors discipline (acting, dance, and psychology) and gender (female vs. male) showed a significant main effect of discipline as well as gender, *F*(2,137) = 7.72, *p* = 0.001 and *F*(1,137) = 5.80, *p* = 0.017, respectively. The interaction of the two factors did not reach significance but showed a very strong trend for a significant interaction, *F*(2,137) = 2.91, *p* = 0.058. Females scored significantly higher than males (Cohen’s *d* = 0.52). Independent *t*-tests showed that the main effect of discipline is based on dancers’ significant lower scores compared to acting and psychology students, *t*(73.15) = 2.31, *p* = 0.023 equal variances not assumed, and *t*(114) = 2.58, *p* = 0.011, respectively (Cohen’s *d* = 0.48, and 0.48, respectively). The RME scores between acting and psychology students did not significantly differ (*p* = 0.927). We then calculated a REML based mixed linear model (MLM) to address our specific interest on discipline and in consideration of the unbalanced design. The MLM with discipline as a fixed factor and gender as a random factor showed a significant effect at the same level as the Univariate ANOVA, *F*(2,139.67) = 4.65, *p* = 0.011. Therefore, albeit the design is unbalanced, the differences in the RME scores across the disciplines are strong enough to show an effect. Bayesian ANOVA with discipline as fixed and gender as random effects shows that this model is 29.16 times more likely than the null model including gender. There is anecdotal evidence for significant effect of discipline on RME scores (BF_10_ = 1.00). The Null model including gender is in weak support of no difference (BF_10_ = 0.034). Bayesian *post hoc* independent *t*-tests showed that there is a strong evidence for a difference between dance and psychology students (BF_10_ = 14.93) and moderate evidence for significant differences between dance and acting students (BF_10_ = 2.98). There is moderate support for no difference between acting and psychology students (BF_10_ = 0.24), all uncorrected.

Applying the same univariate analysis for IRI-PT showed a trend for a main effect of discipline, *F*(2,132) = 2.44, *p* = 0.092. Neither gender nor the interaction between the two factors showed a significant effect (both *p*’s ≥ 0.498). Employing a MLM by removing gender as a random factor from the model, discipline showed the same level of significance, *F*(2,135) = 2.432, *p* = 0.092. Based on our directional prediction for PT (i.e., psychology > dance > acting students), we conducted one-tailed independent *t*-tests. These showed that as predicted, psychology students score significantly higher than acting students, *t*(76) = 1.99, *p* = 0.025 (Cohen’s *d* = 0.52), 75.03 ± 12.07 vs. 69.70 ± 13.00. None of the other comparisons reached significance with dancers scoring 69.37 ± 11.30 [psychology > dance = *t*(116) = 1.46, *p* = 0.073, dance > acting = *t*(78) = 1.08, *p* = 0.142]. Bayesian ANOVA indicates that the data is inconclusive and does not go beyond anecdotal evidence for accepting the null model including gender (BF_10_ = 1.00) or rejecting the H1 hypothesis on the basis of discipline (BF_10_ = 0.558) with the former being 1.8 times more likely. *Post hoc* comparisons confirm that that the supporting evidence for psychology students scoring higher than acting students compared to the alternative hypothesis (acting > psychology) is moderate (BF_10_ = 2.63). The supporting evidence for psychology > dance as well as dance > acting is weak/inconclusive (BF_10_ = 1.07, and BF_10_ = 0.73, respectively).

The univariate analysis for IRI-EC, IRI-PD as well as IRI-FS showed a significant main effect of gender only, *F*(1,132) = 9.27, *p* = 0.003 (Cohen’s *d* = 0.78), *F*(1,132) = 16.66, *p ≤* 0.001 (Cohen’s *d* = 0.84), and *F*(1,132) = 5.87, *p* = 0.017 (Cohen’s *d* = 0.53), respectively. In all subscales, females (*N* = 112) scored higher than males (*N* = 26), IRI-EC (21.61 ± 4.69 vs. 17.85 ± 5.24), IRI-PD (12.71 ± 5.05 vs. 8.78 ± 4.15), IRI-FS (20.96 ± 5.15 vs. 18.23 ± 5.18). Neither discipline alone nor the interaction with gender was significant on IRI-EC (both *p*’s ≥ 0.138), IRI-PD (both *p*’s ≥ 0.304), or on IRI-FS (both *p*’s ≥ 0.267). Neither IRI-PD nor IRI-FS showed a significant effect of discipline after removal of gender from the model; *p* = 0.595 and *p* = 0.160, respectively. Based on the strong indication of gender as a relevant factor in the model of IRI-PD as well as IRI-FS, no further between discipline analyses were performed on these.

However, removing gender from the IRI-EC model, the effect of discipline reached significance *F*(2,134.57) = 3.072, *p* = 0.050. Independent *t*-tests showed that dancers scored significantly lower on IRI-EC than psychology students, *t*(116) = 2.58, *p* = 0.011 (Cohen’s *d* = 0.47). Acting students’ IRI-EC scores were on average lower than those of psychology students, however, the difference did not reach significance, *t*(76) = 1.81, *p* = 0.075. No significant difference between acting and dance students could be observed (*p* ≥ 0.96). Bayesian analysis on IRI-EC showed that the data is nevertheless inconclusive and does not go beyond anecdotal evidence for either accepting the null model including gender (BF_10_ = 1.00) or rejecting the H1 hypothesis on the basis of discipline (BF_10_ = 0.68) with the former being 1.5 times more likely than the latter. Based on the inconclusiveness of either model, no further analyses were conducted.

EQ did not show any significant differences in the ANOVA (all *p*’s ≥ 0.399) nor the MML (*p* = 0.165).

For the correlation analysis (see Table 2), we only included those 85 participants who had data points for all questionnaires, which consisted of 12 acting students (8 females), 25 dance students (21 females), and 48 Psychology students (42 females). The overall scores were REM (26.89 ± 3.34), IRI-PT (18.95 ± 4.89), IRI-EC (21.14 ± 5.13), IRI-FS (20.49 ± 5.38), IRI-PD (11.84 ± 5.21), and EQ (44.73 ± 10.40). The RME scores did not significantly correlate with any of the other empathy measures, except for a significant but weak 1-tailed correlation with EQ, Pearson’s correlation = 0.194, *p* = 0.037. The EQ on the other hand, correlated significantly with all measures except for IRI-PD as indicated in the Table 2. As for the IRI subscales, EC significantly correlated with all other IRI subscales as indicated in the same table, whereas the only other significant but also weak correlation is between PD and FS, with Pearson’s correlation = 0.199, *p* = 0.034.

**TABLE 2:** One tailed Pearson’s correlations between tests.

	**RME**	**IRI-PT**	**IRI-FS**	**IRI-EC**	**IRI-PD**
IRI-EC	0.058	0.368***	0.351***		0.245*
EQ	0.194*	0.323***	0.278**	0.606***	0.027

***p ≤ 0.001, **p ≤ 0.01, *p ≤ 0.05.

## Discussion

We investigated specific facets of empathic abilities in young adults at the starting point of their professional careers in either acting, dancing, or psychology; and who are thus expected to have a preponderance for particular aspects of empathy. Under this premise, we predicted that actors are good at identifying others’ emotional expressions as measured by the Reading the Mind in the Eyes test (RME) but potentially less so in tasks related to other cognitive or affective empathy. In our study, acting students did indeed provide significantly more correct answers in the RME than dance students; and so did psychology students. Additionally, RME scores were dependent on the gender of participants, with females showing a significantly higher emotion recognition performance than males.

Based on numerous evidence for females’ superior empathic abilities that also led to the Empathizing-Systemizing theory of sex differences (Greenberg et al., 2018) and the extreme male brain theory of autism (Baron-Cohen, 2010), we did expect females to show responses that indicate higher empathic skills. The former concept suggests a sex-based classification based on the drive to either empathize (females) or systemise (males), whilst the latter states that males with autism – due to their below average empathy – have an extreme male brain. In our study, females did indeed show overall higher empathy scores than males, which reached significance not only in the RME but also the IRI subscales of Empathic Concern (IRI-EC), Personal Distress (IRI-PD) and the Fantasy Scale (IRI-FS). Notably, the Perspective Taking (IRI-PT) and the E-Drawing Task (EDT) did not show significant gender differences but showed a trend for significant differences across disciplines. Once gender was removed from the IRI-PT model as a between-subjects’ factor, acting students scored significantly lower than psychology students in the cognitive empathy measure of IRI-PT as predicted. Also, the frequency at which acting and psychology students drew the E readable from someone else’s viewpoint (65.5 and 69.8%, respectively) was higher than for dancing students who did so in only 50.7% of the cases.

We suggested that the RME would be particularly suited to measure actors’ prolific empathic abilities and thus predicted that actors would show high scores. Notably, the RME consists of stills taken from actors’ faces in films, thus constitutes a task that is very close to actors’ professional interest. As expected, actors showed significantly higher scores than dancers in the RME, however, there is moderate evidence that the scores do not differ between acting and psychology students. Both acting and psychology students are showing similar proficiency in reading emotions from others’ eyes. The RME asks participants to choose 1 out of 4 terms that best describe the emotional expression of a character’s eyes. As it is important that participants understand the vocabulary at hand they are regularly given access to an accompanying glossary. Nevertheless, verbal skills are evidently a requisite for the RME and several studies show a correlation between drama classes and verbal skills (see Podlozny, 2000). One could therefore argue that our acting and psychology student cohort performed better in RME based on enhanced verbal abilities. We argue that verbal skills do not explain the higher RME scores of our acting cohort. Firstly, it is important to remember that our participants are students in their first year of study. Therefore, our participants can be assumed to have gone through equivalent levels of verbal training. Secondly, whilst it is likely that acting students might have participated in drama classes for some time in advance of their professional training, evidence suggests that neither elementary (Köksal Akyol, 2018) nor middle School drama education (Rickard et al., 2012) were found to significantly enhance verbal skills. In addition, whilst Podlozny’s (2000) meta-analysis showed a strong relationship between verbal skills and engagement in drama classes, the relationship between drama and vocabulary development per see is not reliable. Thirdly, verbal skills components of the RME can be expected to benefit psychology students most profoundly. However, our psychology students did not outperform actors in the RME. In line with behavioral evidence for effects of drama on empathy, emotion regulation and perspective-taking (Winner et al., 2013) and the observation that verbal skills alone predict less than 25% of the variance of RME performance (Peterson and Miller, 2012), we suggest that the acting students’ RME scores were not determined by their verbal abilities *per se* but related to their emotion recognition abilities at least to some degree. This is consistent with the finding of Goldstein and Winner (2010–2011) who observed that verbal ability did not influence REM scores in children participating in acting classes.

Yet as the RME revealed particularly low scores for dance students, it indicates that the RME measures a particular form of empathy, which does not capture other strategies for social interaction. Several studies show support for the assumption that dancers are more focused on the body and its movements than the head. For example, Jola et al. (2011) found that dancers rely more on sensory information from proprioception, even when vision is available. In addition, observational strategies are considered to be individual in nature, with some individuals looking predominantly at upper body parts including the head, and others at lower body parts - depending on the level of expertise as well as the type of sport (Petrakis, 1986). Nevertheless, it is clear that dancers do not frequently look at the face during their learning. In her subsequent dance-specific study, Petrakis (1987) found that undergraduate dance students show a tendency to focus on lower body parts compared to expert dance teachers, who focus more consistently on the upper body parts when watching performers. Yet, overall, the foci on the upper body parts were more prevalently found to consist of eye fixations on the arms than on the head of the model. Further, Stevens et al. (2010) found that dancers’ eye movement patterns indicate that their attention is to predict the movement trajectory of performers’ body, not the head or face. We can thus conclude that psychology and acting students are in general more likely to be focused on the eye region when assessing others’ emotions and thus perform better in this task. Dancers, however, are trained to attend to the body which likely explains the difference in RME scores.

Although dancers’ RME scores are lowest in the present study, this does not imply that dancers would generally be low in their empathic abilities. As the RME is considered to measure cognitive empathy, it is important to highlight that when dancers’ general empathy was measured with the EQ, their scores did not significantly differ from the other two disciplines. In fact, albeit not significant, dance students’ average EQ scores were higher than those of acting students. Moreover, findings by Bonny et al. (2017) suggest that there are differences in cognitive empathy between dancers of different dance styles. The authors found that when controlling for dance styles, dancers with greater Hip hop dance experience have a greater ability to recognize emotions from gaze (RME). Further studies regarding the performing arts found an advantage of acting over dancing: According to Goldstein (2010), children (8–10 years) who did acting, showed higher theory of mind compared to same age children doing dancing. Thus, existing data for dancers’ RME performance remains ambiguous.

As for the IRI, results from all subscales indicated that only Perspective Taking (IRI-PT) and Empathic Concern (IRI-EC) showed moderate differences between the disciplines. The IRI-PT scores indicate that psychology students are more likely to take someone else’s point of view compared to acting and dance students. For IRI-EC we found a significant difference between scores of psychology and dance students, with psychology students showing higher empathic concern for others than dance students. We also found a trend for psychology students scoring better than acting students in IRI-EC. Our findings are in line with previous research, suggesting that acting in young adolescents is not associated with heightened empathy as measured with IRI-EC (Goldstein et al., 2010). Further research on adults comparing actors to psychologists has concluded that actors score lower in IRI-EC (Goldstein et al., 2010), which would indicate that they express less concern for others or are less sympathetic (Davis, 1983). We can imagine that this has benefits for actors’ professional career, as they often need to portray narratives including a variety of positive and negative emotions. Thus, a certain level of dissociation from the character they are playing could help them dealing with negative emotions.

However, a recent study has found contradicting evidence. Panero and Winner (2021) examined the ability of actors to immerse themselves in a role and found that actors scored higher than psychology students in all IRI measures. Whilst these results merit further investigation – it is important to highlight that their design differed from ours. For instance, psychology students were recruited through convenience sampling and incentivised to participate through the use of study credits, whereas actors were recruited through snowball sampling and a financial incentive. The population sampled also differed from our study, whose participants were all at the beginning of their respective careers. Moreover, in the Panero and Winner study, psychology students were recruited from a narrower age range (18–22) than the actors (18–30). Lastly, it’s possible that cultural differences, such as training styles, were partly responsible for the difference in results. The combination of these factors might have caused the apparent contradiction between the empirical evidence of both studies. Consequently, these factors, together with the relationship between acting and IRI scores, would benefit through further investigations.

Another notable result in our study is the lack of significant differences between the disciplines in the other IRI subscales, the IRI-FS and the IRI-PD, which is surprising. The fantasy scale seems to measure traits typical to actors, such as an inclination to empathize with fictional characters (Kaplan and Iacoboni, 2006). Previous research has suggested a trend for higher neuroticism in actors (Nettle, 2006), which supports Davis’ (1983) association between fantasy scale and shyness, loneliness, and social anxiety, especially among males. However, Guilera et al. (2019) did not find a significant correlation between IRI-FS and neuroticism. To clarify why our IRI-FS and IRI-PD scores do not show any discipline differences, we compared our IRI subscale scores with those from the general population. Whilst our scores are in a comparable range to those originally reported by Davis (1980, 1983) but only separately for males and females; one-sample *t*-tests of our sample’s means in comparison to mean scores across gender, available from a larger sample of 651 participants pooled from eight other studies (De Corte et al., 2007), showed that our cohort (*N* = 138) scored significantly higher in IRI-FS [20.45 ± 5.24 vs. 16.48 ± 5.91, *t*(8.89), *p* ≤ 0.001], IRI-PT [18.52 ± 4.91 vs. 17.29 ± 4.30, *t*(2.94), *p* = 0.004], and IRI-EC [20.90 ± 5.00 vs. 18.05 ± 4.23, *t*(6.69), *p* ≤ 0.001] but not IRI-PD [11.93 ± 5.14 vs. 11.92 ± 4.87, *t*(0.017), *p* = 0.986]. We can therefore conclude that acting, dancing, and psychology students do stem from a similar cohort that does engage in fantasizing, perspective taking, and empathic concern over and above the general population. This is in line with the findings for perspective taking and empathic concern by Gujing et al. (2019), which showed that dancers self-reported empathic abilities are higher compared to a general cohort. In contrast, we did not find that our cohorts’ personal distress scores differ from the general population. The level of personal distress our participants report to experience in response to others’ disadvantages does indeed seem to be specific for gender only and not affected by other characteristics, such as engagement in the performing arts or psychology.

The EDT, on the other hand, showed that half of the dancers draw the E from their own perspective. The relationship between EDT, perspective taking and prosocial behavior (Adida et al., 2018) would suggest that psychology students, whose academic performance was found to improve with prosocial behavior (Hassall et al., 2015), should be the most oriented toward taking others’ perspective. The relationship between the other two disciplines and prosocial behavior is, however, understudied. Actors, who also showed a tendency to take others’ perspective compared to dancers, might also be prosocially inclined. The lack of a tendency toward other-centered perspective by the dancers could be explained by dancers’ inherent focus on their own body and their own performance. While actors and psychologists are more likely reliant on others with regards to their own performance, dancers need to master their own point of view, first and foremost. Besides, their form of communication relies much more on the spectator taking the dancer’s perspective. It is likely, however, that as with other empathy measures, that there are notable differences between different dance styles.

EQ scores are comparable across all three participant groups and correlated moderately with three of the IRI subscales (IRI-PT, IRI-EC, and IRI-FS), yet not with IRI-PD. EQ scores further showed a weak correlation with the RME data. Given that EQ predicts subjective kinaesthetic feelings (Seiryte and Rusconi, 2015), and that actors have shown high scores in the EQ task in Baron-Cohen and Wheelwright’s (2004) study, it is surprising to see that there was no effect of profession on EQ scores in our study. Though this may be a by-product of the subjective nature of the measure (Baron-Cohen and Wheelwright, 2004). Furthermore, the fact that our EQ scores did not correlate with PD, and that the PD scores of our cohort are not different from the general population, the correlation found by Khajeh et al. (2014) between EQ and psychological wellbeing is of relevance. It would be of interest to further investigate this matter with regards to the relationship between PD and wellbeing. Finally, while EQ was shown to predict performance in a face perception task (Penton-Voak et al., 2007), this did not result in an increased RME score for participants high in EQ. Perhaps individuals with high EQ focus on other parts of the face instead of the eyes and are therefore disadvantaged when only the gaze is presented.

It is important to understand that our study cannot distinguish whether individual differences in empathic abilities are based on training, education or whether they denote an inborn characteristic. Moreover, whilst it would be beneficial to identify if specific facets of empathy are inherited or acquired through training, this was not the aim of our study. We set out to identify (1) whether different facets of empathy are prevalent at early stages in three self-assigned student groups and (2) whether certain empathy tests are susceptible to these differences. We argue that it is not feasible to match the physical and theoretical training for these disciplines, since they inherently follow specific professional trajectories: Actors, dancers, and psychologists require different levels of preparation. For example, dancers often start their training as early as the age of five in order to gain sufficient experience to start full time professional training. Psychology students, however, might have had personal life experiences that activated their level of social responsibility wanting to help others deal with their emotions or that developed their need to study psychology in order to be in a better position to deal their own emotions. Finally, acting students might have practiced their social interaction skills through everyday life improvised actions.

Asking participants to complete four empathy surveys as part of one study can lead to response fatigue. We found that of those participants who completed the first empathy measure, over 2/3rd of participants (72%) completed the full set of questionnaires, which is a good adherence. Nevertheless, we suggest that future research that aims to compare measures of empathy facets across a set of questionnaires would benefit from evaluating the use of shorter versions, such as the B-IRI (Ingoglia et al., 2016) or the EQ-Short (Wakabayashi et al., 2006). In line with this, it is notable that our groups were unbalanced in numbers with the smallest being the acting students. This is unfortunate yet not surprising, as actors are generally underreported in empirical research, compared to other performing art cohorts. We remain hopeful that this will change with the recently increased efforts in experimental studies on actors’ perception and cognition (see Noice and Noice, 2013; Lippi et al., 2016).

Another limitation is that empathy is considered to consist of three components, i.e., cognitive, affective, and behavioral (Lam et al., 2011). Whilst we did include two indirect measures of empathy and ensured that we test for cognitive and self-reported affective facets, we did not actually measure participants empathic or pro-social behavior. Notably, only a small number of studies have done so (e.g., Chen et al., 2010; Martínez-Velázquez et al., 2020). It would be good to get a better understanding of the relationship between different empathy facets for different cohorts, as well as the effects of their professional interests on actual pro-social behavior.

Probably the most stringent conclusion of our findings is the support for the argument that actors, dancers, and psychologists do *perform* in everyday life. This is indicated for example through this cohorts’ IRI scores which are higher compared to the general population in all subscales but personal distress. Hence, they have learnt about empathy and can act upon it (i.e., see also Lam et al., 2011), without above average personal suffering. One way to achieve this can be through creating a mental state for performance that has a high level of constructive conscious control, as in the Alexander technique, practiced by many actors and dancers (Alexander, 2004). As our cohort was at the start of their training, it would be of great interest to explore whether such a conscious control is more prevalent in individuals that seek a profession which requires specific aspects of empathy, such as actors, dancers, and psychologists.

Yet, it is possible that some findings are based on how well the empathy measure matches the actual practice of the discipline. For example, actors might have high RME scores because the task is close to their everyday interests rather than their cognitive empathy skillsets. This is supported to some extent in that they do not score higher than psychology students on other cognitive empathy scores, such as perspective taking. Similarly, dancers are internally focused and their empathy scores are therefore less prone to cognitive empathy (RME, EDT, and IRI-PT) but also lower in affective empathy (IRI-EC). This could be interpreted in that either the tests were least suitable in identifying dancers’ empathic abilities due to their focus on faces and their static characteristics, or because dancers are in fact not very empathic. The latter would have significant implications since many studies used dance as a form of intervention to enhance empathic skills. Notably though, whilst several studies have used dance training with the aim to enhance empathic abilities (e.g., Zazulak et al., 2017; Mastrominico et al., 2018), there is only sparse evidence for dancers having better empathy skills or for the effects of dance interventions to increase empathy. In fact, several studies did not find evidence for enhanced empathy: Mastrominico et al. (2018) found no effect of dance intervention through mirroring or imitation/synchronization on adults’ empathic abilities with ASD and Zazulak et al. (2017) found no effect of mindfulness dance intervention in medical students’ empathic ability scores. Moreover, whilst Koehne et al. (2016a) found that a dance intervention focusing on interpersonal movement imitation and synchronization, enhanced facets of empathy skills in adults with ASD with participants showing enhanced emotion inference abilities, the authors did not find an increase in empathic feelings. Henceforth, to date, dancers might have increased internal awareness (Jola et al., 2011; Christensen et al., 2018), yet empathy enhancements that also require increased external awareness in response to dance training is limited to anecdotal evidence (Hahn, 2015; Gallagher and Flint, 2016). One explanation could be that different dance styles are linked with different levels of inter-personal synchronization, which is considered an important aspect of empathy (Koehne et al., 2016b). It is thus important that first, we understand better which kind of dance practice increases empathic skills before it is employed with that aim. Furthermore, our findings suggest that acting interventions may be more beneficial than dance interventions: our acting cohort showed higher levels of emotional recognition than dancers did while also reporting empathic concern above the average population, without getting too distressed about it. Therefore, acting training can be considered to build emotional resilience; or in other words, to enhance the ability recognize another’s emotional expression with some feeling for them, but to do so from an emotional distance. The ability to regulate one’s own emotions in social-interactions is a crucial ability in general (Thompson et al., 2019) and for actors in particular (Berceanu et al., 2020). Importantly, individuals who show an interest and engagement in acting practice do already show higher cognitive empathy skills (here RME and EDT) at the start of their career.

To conclude, whilst acting, dance, and psychology students score higher than the general population in all IRI subscales, including empathic concern but not personal distress, shows that these cohorts ‘know’ how to feel yet manage to keep an emotional distance. This in turn suggests that those individuals who study acting, dance, or psychology have empathic tools available at the start of their training that allows them to experience empathic concern without strong emotional distress. However, as our acting students showed significantly higher RME scores than the dancers and are significantly more often accounting for the other’s perspective (according to the EDT), future studies should explore whether acting training might be better suited for developing cognitive empathy or Theory of Mind than dance. Nevertheless, the impact of theoretical empathy training should not be underestimated, considering that psychology students’ performance in the RME and EDT did not significantly differ from actors. We did not expect psychology students to perform so well across the different empathy tests. However, we did not measure general levels of intelligence, which may have had an impact on these scores. Importantly, since empathic concern measures a person’s ability to “tune in” to other people’s emotional states, a high sensitivity would normally become overwhelming. Whether it is through indirectly trained resilience, general levels of intelligence, or an ability to compartmentalize, actors, dancers, and psychologists might be able to moderate this often-overwhelming sensitivity, and thus do not report above average personal distress. Future research should investigate whether people from the general public, who do not undergo any sort of empathy training, but score high on affective and cognitive facets of empathy, do experience high personal distress or not.

## Data Availability Statement

The dataset generated for this study can be found in the Open Science Framework (https://osf.io/mjkfh/) with accompanying codebook.

## Ethics Statement

The studies involving human participants were reviewed and approved by School of Health and Social Sciences Research Ethics Committee (today School of Applied Sciences). Participants could only take part in this online study if they provided informed consent to participate.

## Author Contributions

CJ designed and implemented the study, analyzed the data, and wrote the manuscript. IS, RSL, and TR contributed to the data collection and analysis and the writing of the manuscript. IS, RSL, and CJ provided critical feedback on the manuscript and aspects of the rationale. All authors contributed to the article and approved the submitted version.

## Conflict of Interest

The authors declare that the research was conducted in the absence of any commercial or financial relationships that could be construed as a potential conflict of interest.
